# Gender differences in paid work over time: Developments and challenges in comparative research

**DOI:** 10.1371/journal.pone.0322871

**Published:** 2025-05-14

**Authors:** Lena Hipp, Kristin Kelley

**Affiliations:** 1 University of Potsdam, Potsdam, Germany; 2 WZB Berlin Social Science Center, Berlin, Germany; Bar-Ilan University, ISRAEL

## Abstract

This paper examines gender differences in paid work over time and illustrates the pitfalls encountered by any comparative research that only considers either labor force participation rates or average working hours. To do so, we analyze harmonized survey data from Europe and the United States from 1992 to 2022 (N = 43,283,172) and show that more progress was made in closing gender gaps in labor force participation rates than in working hours. In most countries, women’s labor force participation rates increased considerably, but their average working hours decreased, whereas both men’s labor force participation rates and average working hours decreased or stagnated (but nonetheless still remained much higher than women’s). We show and argue that these countervailing trends in working hours and labor force participation rates make it difficult to paint a coherent picture of cross-national differences in women’s and men’s paid work and of changes over time. In response, we propose “work volume” as a supplementary or alternative measure for any type of comparative research. Work volume records zero working hours for nonemployed individuals and thus allows straightforward comparisons between women’s and men’s (or any other groups’) involvement in paid work. Using the proposed work volume measure, we show that gender gaps in paid work decreased over time, but that even in 2022, men’s involvement in paid work remained considerably higher than women’s—with gender gaps being lowest in the Scandinavian and the former Communist countries.

## Introduction

How have gender differences in paid work evolved between countries and over time, and what do researchers and policy makers need to consider when engaging in such comparisons? Research addressing the first question has often used labor force participation (LFP) rates—that is, the proportion of the population that is either employed or actively seeking employment [1]. However, there is a problem with the use of LFP rates in comparative research, as they overlook the large cross-country differences in the weekly working hours of employed people. The other alternative—using average working hours—is also not without problems, as it overlooks the proportion of the population that is not working. The following example illustrates this dilemma: In 2022, LFP rates of prime-working-age women (25–54 years) were similar in Switzerland and Portugal (86 and 89%) [2], but the average weekly working hours of employed women differed significantly between the two countries (32 hours in Switzerland and 39 hours in Portugal) [3]. Likewise, women’s average working hours were similar in Austria and Italy (32 and 33 hours) [3], but women’s labor force participation was much higher in Austria than in Italy (87 and 69%) [2]. It is hence essential to jointly examine LFP rates and average working hours when examining group differences in paid work. After all, the overall economic well-being of any group within a country is also shaped by both individuals’ labor force participation and their working hours [4,5].

To systematically examine gender differences in paid work and trace developments between countries and over time, we analyze harmonized labor force data from Europe (EU-LFS) and the United States (IPUMS-CPS). First, we present the empirical developments for 32 countries since the early 1990s. Second, we show that the two commonly used measures of involvement in paid work—LFP rates and average weekly working hours—do not necessarily change in the same direction. Any comparison across countries, over time, or by demographic characteristics should therefore rely on both LFP rates and average working hours—either in separate analyses or with a combined measure. Third, for this combined measure, we propose what we call a “work volume” measure that captures the average working hours of *all* individuals by recording hours of zero for those who are not working, regardless of whether they are unemployed, on some form of leave, or not in the labor force at all. Depending on the research question, work volume can be used as an alternative or supplementary measure to LFP rates and average working hours.

The suggested work volume measure is particularly advantageous for comparative research. By comparing work volumes, researchers can capture involvement in paid work by the entire population of interest, without needing to exclude certain groups of women and men (e.g., the unemployed, part-time workers, parents on family leave) or defining a threshold for full-time employment for countries with different definitions of full-time work. In short, work volume is well-suited to macrolevel analyses that seek to assess (gender) differences in paid work—both from a cross-country comparative and longitudinal perspective.

## Litervature review

A central thread in the scholarship on work and gender concerns women’s involvement in paid work. This research seeks to understand whether progress toward gender equality has stalled, is uneven, or is still underway [6–8]. Scholars have thus examined changes in women’s paid work over time within countries and across countries by analyzing women’s employment and labor force participation rates [6,9]. For example, much attention has been devoted to women’s stagnant labor force participation rates in the United States [10–14], which steadily increased until 1990, but stalled through the 1990s and 2000s. Similar trends can also be observed in some European countries [15,16].

In addition, research has also examined women’s and men’s working hours as a means of evaluating labor market inequalities [17]. This is an important measure because working full-time is associated with greater financial autonomy. Among employed people, part-time work and interruptions to employment reduce earnings, decrease pension payments, and increase the dependence on a partner or welfare state support [5,18,19]. In cross-country comparisons, researchers have found considerable variation in working hours among employed women based on their parental status [20,21]. The time spent providing childcare, caring for other relatives, and performing other unpaid tasks often make it impossible for women to work full time, even when they have access to a well-developed childcare infrastructure [7,22,23].

Structural, cultural, and compositional characteristics explain variation in paid work between countries and socio-demographic groups. Economic conditions, such as the level of economic development, unemployment rates, and labor market regulations influence whether, to what extent, and which groups of people pursue paid employment [7,21,24–26]. Research has also indicated that gender role norms, attitudes towards maternal employment, and work-family policies explain differences in women’s and men’s paid employment [7,21,24–26]. Lastly, compositional characteristics also affect variation in labor force participation rates and working hours. For instance, when comparing women’s involvement in paid work between countries and across time, parental and partnership status, number and age of children in the household, or partners’ working hours and involvement in unpaid work have been found to influence both women’s employment and their working hours [27–30].

There is a key challenge for any type of labor market comparison—whether between genders, countries, time points, or any other quantities of interest: Considering either labor force participation rates or working hours in isolation may lead to bias results and we may draw inaccurate conclusions about the enabling or constraining effects of macro and micro-level characteristics. For instance, in the Netherlands and Sweden, women’s employment rates are high (around 82 percent), but employed women work part-time to different extents. In Sweden, 12.9% of all employed women work part-time, while this proportion amounts to more than 50% in the Netherlands [31–33]. Mere comparisons of LFP rates—over time, across countries, or between socio-demographic groups—thus mask differences in working hours and hence also in the extent to which different socio-demographic groups earn decent wages and benefit from social protections. Likewise, when comparing working hours, researchers tend to limit their comparisons to employed people to derive comparable groups (e.g., dual-earner couples) [17,27]; in the process, they do not consider LFP rates or those who are not in the labor force at all.

Because relying on one measure can lead to incomplete conclusions about the role of both macro and micro-level explanations for (gendered) labor market inequalities, scholars have begun to check whether results generated from women’s LFP rates are consistent with those with women’s working hours [21,26,34]. Alternatively, they have limited their analyses to employment rates of women who work full-time [15,35,36], which is also not without problems given that definitions of “full-time” work differ across countries and studies [34–37] and that the insights generated from this studies possibly ignores meaningful changes and differences among those who work less than “full-time.”

## Methods

To conduct research that is comparable across countries and over time, we propose researchers use a new measure of women’s involvement in paid work, which we call “work volume.” The measure captures the average hours of paid work per week for all women and men of working age and records hours at zero for women and men who are not working, regardless of whether they are unemployed, non-employed, or on parental leave. Work volume hence combines information about employment rate and weekly working hours. Recall that increases in labor force participation inflate perceptions of change in countries where many women enter the labor force, even though they may only work a few hours per week. Measures centering on working hours inflate perceptions of change in countries if increases or decreases in hours are only observed in the select group of already employed women or men. By contrast, work volume captures the overall contribution of women and men to paid work and also the average visibility of women and men in the labor force within a particular country. Thus, comparing work volume could give us greater insight into public perceptions of women’s and men’s overall attachment to paid work. Additionally, work volume is a standardized measure that does not rely on defining how many hours count as “full-time” or require researchers to exclude certain groups of women and men, e.g., the unemployed or part-time workers [34].

Next, we illustrate the utility of the proposed “work volume” measure and explore whether it offers new insights into the evolution of gender gaps in paid work across countries and over time. To this end, we analyze differences in LFP rates, average weekly working hours, and our suggested work volume measure. To do so, we harmonized individual-level data from the U.S. Current Population Survey [IPUMS-CPS, 38] and the EU Labor Force Survey (EU-LFS) for the years 1992–2022 [15,25,39], relying on common definitions provided by the International Labor Organization (ILO) to ensure data comparability.

We measure LFP rates as the percentage of the prime-working-age population (25–54 years old) who currently work for pay at least one hour per week, are currently unemployed and actively looking for employment, or are on a temporary leave of absence from work (e.g., parental leave) [40]. We measure average weekly working hours as respondents’ mean actual hours in their first and second job (if they have one) during a reference week. Using actual working hours during a reference week rather than usual working hours leads to more accurate comparisons because a) the calculations are based on working hours from all jobs and not only the main job, b) respondents do not have to calculate averages for a “usual” week, and c) differences in national holidays and durations of leave are taken into account. The work volume measure is similar to working hours but also includes people outside of the labor force, unemployed, or on leave with zero working hours. Thus, no respondents are excluded.

We examine descriptive values for each of the three paid work measures for 32 countries from 1992–2022 by respondent gender. All analyses are restricted to the civilian, prime-working-age population (25–54 years). We employ the age restriction to account for variation in education and retirement systems across countries and over time [41]. Our final analytic sample includes 43,283,172 individuals in 864 country-years. As our focus is on long-term trends in men’s and women’s involvement in paid work, we do not discuss COVID-19-related changes in LFP rates and working hours in great detail, as they have been discussed extensively elsewhere [42–45].

**Fig 1:**
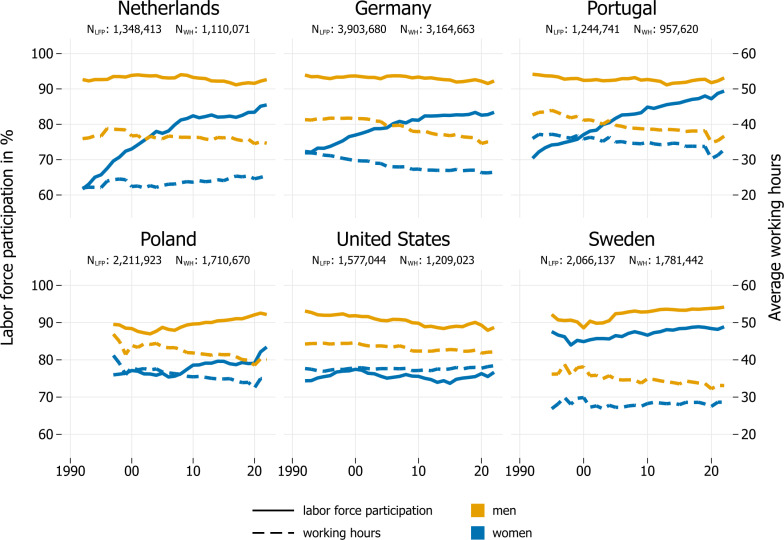
Labor force participation rates and average working hours in six focal countries, 1992–2022. Note: Figure is based on individuals aged 25-54 years. Data for Sweden are available since 1995 and for Poland since 1997. Fig A1 in the Online Appendix shows trends for all countries and measures.

**Fig 2:**
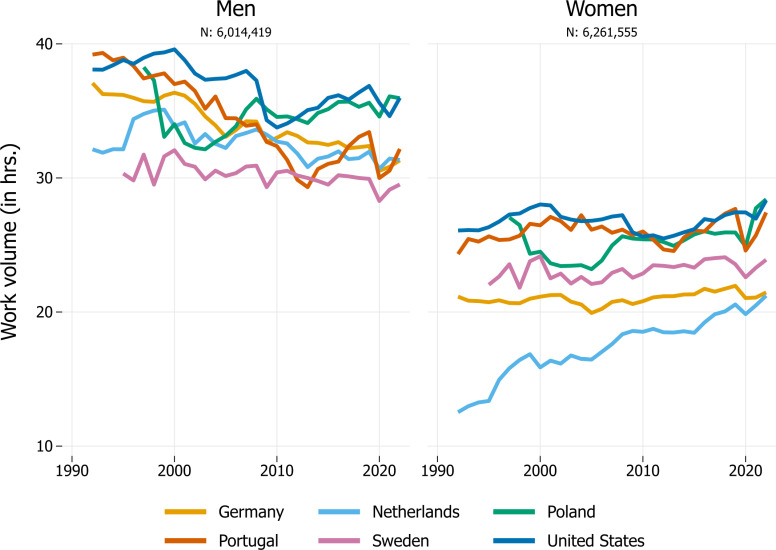
Trends in average work volume (in hrs.) in six focal countries, 1992–2022. Note: Figure is based on individuals aged 25-54 years. Data for Sweden are available since 1995 and for Poland since 1997. Fig A1 in the Online Appendix shows trends for all countries.

In the following, we start by graphically presenting differences in developments in LFP rates and average working hours for six focal countries (Fig 1) to illustrate the challenges in comparing involvement in paid work across both countries and time. After establishing the usefulness of the work volume measure (Fig 2, again for six focal countries), we then turn to the detailed summary characteristics for the entire sample of countries (Table 1). Graphical illustrations for all countries in our sample are available in the Fig A1 in Appendix.

**Table 1:** Gender gap measures by country.

			Women			GenderGap(Men-Women)		
Regime	Country	First/Last Year	LFP %	Hours h/week	Work Volume h/week	LFP %	Hoursh/week	Work Volumeh/week
	Austria	1995	73.34	34.38	23.35	19.92	7.49	14.29
		2022	86.63 **↑**	25.66 **↓**	21.29 **↓**	5.98 ↓	9.13**↑**	9.49 ↓
	Belgium	1992	64.34	33.11	19.35	27.76	8.32	17.13
		2022	81.94 **↑**	29.32 **↓**	22.88 **↑**	8.23 ↓	7.02 ↓	8.21 ↓
	Switzerland	1996	76.07	26.73	19.34	21.31	14.82	19.80
		2022	86.07 **↑**	28.96 **↑**	23.74 **↑**	7.82 ↓	9.84 ↓	11.31 ↓
	Germany	1992	72.32	31.93	21.15	21.61	9.38	15.92
		2022	83.38 **↑**	26.47 **↓**	21.46 ~	8.87 ↓	8.58 ↓	9.85 ↓
	France	1992	74.72	31.41	20.81	20.42	7.96	14.12
		2022	84.34 **↑**	28.98 **↓**	22.89 **↑**	7.91 ↓	5.33 ↓	6.79 ↓
	Luxembourg	1992	54.92	34.25	18.27	39.98	6.14	19.60
		2022	86.92 **↑**	30.04 **↓**	24.88 **↑**	5.80 ↓	5.91~	7.07 ↓
	Netherlands	1992	61.69	22.02	12.53	30.96	13.93	19.60
		2022	85.48 **↑**	25.51 **↑**	21.22 **↑**	7.16 ↓	9.13 ↓	10.13 ↓
**Continental/ Conservative**	**Ø** *Change*	**Last**	**84.36** 7 ↑	**27.08** 2 ↑ 5 ↓	**21.65** 5 ↑ 1 ↓ 1 ~	**7.79** 7 ↓	**8.44** 5 ↓ 1 ↑ 1~	**9.51** 7 ↓
	Ireland	1992	49.09	30.69	12.91	42.30	12.40	20.90
		2022	80.42 **↑**	29.01 **↓**	22.15 **↑**	11.58 ↓	8.28 ↓	10.64 ↓
	Malta	2009	48.87	32.65	14.98	45.05	8.63	21.89
		2022	83.95 **↑**	32.28 ~	26.44 **↑**	12.29 ↓	4.35 ↓	7.92 ↓
	UK	1992	73.56	26.50	18.08	20.51	14.42	16.45
		2019	81.90 **↑**	29.09 **↑**	23.11 **↑**	10.70 ↓	9.32 ↓	11.47 ↓
	US	1992	74.39	37.68	26.08	18.72	6.58	12.01
		2022	76.68 **↑**	38.44 **↑**	28.32 **↑**	12.01 ↓	3.69 ↓	7.65 ↓
**Anglo-Saxon/** **Liberal**	**Ø** *Change*	**Last**	**78.42** 4 **↑**	**34.58** 2 ↑ 1 ↓ 1 ~	**25.30** 4 **↑**	**13.16** 4 ↓	**5.17** 4 ↓	**9.03** 4 ↓
	Bulgaria	2000	78.93	39.16	25.53	5.47	1.73	2.80
		2022	82.53 **↑**	37.40 **↓**	29.42 **↑**	6.50**↑**	1.49~	3.35**↑**
	Czech Republic	1997	82.10	38.86	30.18	12.99	6.15	11.31
		2022	81.60 ~	32.57 **↓**	25.87 **↓**	14.66**↑**	5.45 ↓	10.16 ↓
	Estonia	1997	84.78	39.53	30.27	7.17	3.44	5.13
		2022	87.41 **↑**	30.71 **↓**	25.54 **↓**	6.54 ↓	4.44**↑**	5.67**↑**
	Hungary	1996	68.33	37.53	23.61	17.56	4.75	9.25
		2022	87.73 **↑**	33.16 **↓**	28.17 **↑**	6.58 ↓	4.72~	6.41 ↓
	Lithuania	1998	87.04	38.17	29.34	5.19	4.27	4.24
		2022	88.97 **↑**	33.69 **↓**	28.49 ~	2.27 ↓	3.83~	3.72 ↓
	Latvia	1998	83.92	41.01	30.03	7.15	2.70	4.45
		2022	84.41 ~	34.26 **↓**	27.03 **↓**	6.52 ↓	4.02**↑**	4.84~
	Poland	1997	75.98	41.19	27.05	13.55	5.70	11.21
		2022	83.45 **↑**	35.08 **↓**	28.41 **↑**	8.72 ↓	5.02 ↓	7.53 ↓
	Romania	1997	79.82	39.90	30.27	12.72	3.97	8.63
		2022	72.44 **↓**	38.04 **↓**	26.43 **↓**	18.84**↑**	1.74 ↓	8.05 ↓
	Slovenia	1996	82.64	38.26	30.06	7.95	4.19	6.20
		2022	90.51 **↑**	32.27 **↓**	28.10 **↓**	4.58 ↓	4.30~	5.56 ↓
	Slovak Republic	1998	81.30	38.52	27.77	12.40	3.56	7.90
		2022	86.27 **↑**	30.42 **↓**	24.62 **↓**	7.01 ↓	6.33**↑**	7.78~
**CEE/ Former Communist**	**Ø** *Change*	**Last**	**83.37** 7 ↑ 1 ↓ 2 ~	**34.34** 10 ↓	**26.96** 3 ↑ 6 ↓ 1 ~	**8.76** 7 ↓ 3↑	**3.81** 3 ↓ 3 ↑ 4~	**6.33** 6 ↓ 2 ↑ 2~
	Cyprus	2000	68.57	35.27	22.57	26.73	5.98	15.58
		2022	84.88 **↑**	32.73 **↓**	25.80 **↑**	9.34 ↓	4.17 ↓	7.23 ↓
	Greece	1992	51.63	39.25	18.26	42.04	5.45	22.06
		2022	77.41 **↑**	34.92 **↓**	22.47 **↑**	15.53 ↓	6.96**↑**	12.81 ↓
	Spain	1992	50.43	34.13	13.32	42.56	4.64	18.74
		2022	83.24 **↑**	29.79 **↓**	21.24 **↑**	8.37 ↓	5.04**↑**	7.35 ↓
	Croatia	2002	73.72	37.40	23.33	13.20	3.10	8.18
		2022	82.53 **↑**	33.16 **↓**	25.37 **↑**	6.92 ↓	3.09~	5.18 ↓
	Italy	1992	52.65	33.08	15.67	39.42	6.71	19.39
		2022	68.54 **↑**	29.82 **↓**	18.41 **↑**	20.03 ↓	7.43**↑**	12.30 ↓
	Portugal	1992	70.39	35.99	24.32	23.76	6.65	14.88
		2022	89.39 **↑**	32.84 **↓**	27.43 **↑**	3.74 ↓	3.79 ↓	4.74 ↓
**Southern**	**Ø** *Change*	**Last**	**79.71** 6 **↑**	**31.51** 6 **↓**	**22.30** 6 **↑**	**10.95** 6 ↓	**5.26** 2 ↓ 3 ↑ 1~	**8.12** 6 ↓
	Denmark	1992	87.40	29.88	23.69	6.61	8.34	9.44
		2022	85.25 **↓**	28.13 **↓**	23.10 **↓**	4.96 ↓	5.80 ↓	6.41 ↓
	Finland	1995	82.41	30.45	21.38	5.90	6.69	5.31
		2022	86.79 **↑**	28.58 **↓**	23.37 **↑**	2.60 ↓	4.43 ↓	4.40 ↓
	Iceland	1995	86.68	32.16	26.68	9.62	17.34	19.13
		2022	86.85 ~	27.88 **↓**	23.42 **↓**	5.97 ↓	5.99 ↓	7.01 ↓
	Norway	1995	81.08	30.94	23.34	10.16	9.92	12.06
		2022	83.85 **↑**	26.84 **↓**	21.98 **↓**	5.36 ↓	4.91 ↓	5.64 ↓
	Sweden	1995	87.60	26.87	22.04	4.59	9.29	8.29
		2022	88.88 **↑**	28.67 **↑**	23.90 **↑**	5.28**↑**	4.43 ↓	5.61 ↓
**Scandinavian/** **Social-democratic**	**Ø** *Change*	**Last**	**85.35** 3 ↑ 1 ↓ 1 ~	**28.03** 1 ↑ 4 ↓	**22.65** 2 ↑ 3 ↓	**5.58** 4 ↓ 1↑	**5.39** 5 ↓	**6.10** 5 ↓
**Overall country Ø** *Change over time*		**Last**	**83.77** 27 ↑ 2 ↓ 3 ~	**31.08** 5 ↑ 26 ↓ 1 ~	**24.59** 20 ↑ 10 ↓ 2 ~	**8.40** 28 ↓ 4↑	**5.56** 19 ↓ 7 ↑ 6~	**7.57** 28 ↓ 2 ↑ 2~

As the focal countries for the graphical illustrations, we chose Germany, the Netherlands, Poland, Portugal, Sweden, and the United States. These countries represent different geographical regions and welfare regimes [46,47]. It is important to cover different regimes, because they vary in their reliance on the family, the state, or the market to provide welfare [48,49], which in turn, affects women’s and men’s participation in paid work [46,50–53]. As there is also within-variation in welfare state regimes and regional families of countries, we include two countries from the continental European, conservative welfare state family—Germany and the Netherlands. These two countries resemble each other on relevant economic, policy, and cultural dimensions but nonetheless vary considerably in the extent to which women and men work in part-time jobs: In the Netherlands, almost 40% of the work is in part-time employment, and more than 60% of these are female; in Germany, part-timers make up less than 30% of the labor force and less than 50% of these are women [50].

## Results

Fig 1 illustrates how LFP rates and average working hours of the focal countries have evolved over time. Since the early 1990s, there have been considerable increases in women’s LFP rates in the Netherlands (+24 pp), Portugal (+19 pp), and Germany (+11 pp), a slight increase in Poland (+7 pp since 1997), and stalls in the United States (+2 pp) and Sweden (+ 1 pp pp; data for Sweden are only available from 1995).

Interestingly, changes in average working hours do not mirror those in LFP rates. Although women in Germany, Portugal, and Poland had increasing LFP rates, they had decreasing average working hours over time (-5.5 hours in Germany, -6.1 in Poland, -3.2 in Portugal). However, in the Netherlands, which also had increasing LFP rates, women’s average working hours increased by 3.5 hours (the largest increase of all countries in the sample). In Sweden, where LFP rates stagnated, there was an increase in women’s average working hours (+1.8 hours). There has been little change (+0.8 hours) in the high average working hours of women in the United States (38 hours), the only country in the sample in which both indicators plateaued.

In contrast to the large variation in LFP rates and working hours among women during the observation period, men’s LFP rates in the six focal countries remained high and changed little since the 1990s. The largest decrease in LFP rates of any country was in the United States (-4.5 pp). Men’s average working hours decreased in every country over the observation period, although to different degrees (from -1.3 hours in the Netherlands to-6.2 hours in Germany).

To assess gender inequalities across countries, we now turn to the gender gaps in LFP rates and working hours. Women have had lower LFP rates and lower working hours than men in all countries, but the size of these gaps varies. While the gender gap in LFP rates in 2022 suggests higher gender equality in the Netherlands (gap of 7 pp) than in the United States (gap of 12 pp), the United States are much more gender equal when considering the gap in average working hours (less than four hours in the United States vs. more than 9 hours in the Netherlands). Hence, while the gap in one indicator may suggest higher equality in one country, the gap in another indicator may favor a different country.

Given these complex patterns, it is difficult to order countries by labor market equality. Investigating LFP rates or average working hours *alone* would present a distorted picture. However, as illustrated by comparing gender gaps in both indicators for the Netherlands and the United States, even when we examine both LFP rates and average working hours, it is not entirely clear how countries rank in terms of gender (in)equality. Therefore, we use our suggested summary indicator—the differences in men’s and women’s average work volume over time—to provide a better starting point for conversations about overall labor market performance and gender inequalities in paid work.

Fig 2 reports women’s and men’s work volume across the six focal countries. Broadly speaking, the figure shows three trends: First, men’s average work volume has decreased over time (particularly in Portugal and Germany). Second, women’s average work volume has either increased (e.g., the Netherlands), remained relatively stable (e.g., Germany), or fluctuated, with no evidence of long-term change (e.g., Poland). Third, gender gaps in work volume have declined in the six focal countries, although to different degrees and due to different developments. The decline in the Netherlands, for example, is attributable to women’s increase in work volume, but the decline in Germany is due to men’s decrease in work volume. Despite their declining gender gaps, Germany and the Netherlands continue to have some of the largest gender inequalities in work volume (around 10 hours). The gaps in Portugal (4.7 hours) and Sweden (5.6 hours) are among the smallest.

In a last step, we expand our analyses to the entirety of countries in our sample (Table 1) and summarize the data for the first and the last survey year. In addition to showing women’s LFP rates, working hours, and work volume, we also display the gender gaps in each of these three measures: Upward arrows indicate increases of > 0.5 in women’s LFP rates, average working hours, and work volume over time and increases in gender gaps in each of these three measures, while downward arrows indicate declines of < 0.5. The countries are clustered by geographical region/type of welfare state regime [47].

Overall, Table 1 shows that across all countries in our sample, the average gender gap in work volume amounts to 7.57 hours per week—ranging from less than 4 hours in Bulgaria and Lithuania to around 12 hours in Italy, Greece, and the UK—and has decreased in 28 out of the 32 countries (it increased in Bulgaria and Estonia and stayed the same in Latvia and the Slovak Republic). The overall trend towards a narrowing work volume gap is related to two developments: rising labor force participation rates among women in almost all countries (exceptions are the Czech Republic, Denmark, Iceland, Latvia, and Romania) and men’s decreasing average working hours (which can be seen indirectly in Table 1 through gender gaps in average working hours). Even though women’s average working hours decreased in 26 out of 32 countries, gender gaps in working hours also decreased in 19 countries, which means that men have reduced average working hours to even larger extents than women.

The clusters with the smallest gender gaps in work volume in are the Central and Eastern European (CEE) countries, with their communist legacy of promoting women’s economic independence, and the social-democratic Scandinavian countries, where women’s involvement in paid work has been supported by universal access to childcare and egalitarian family policies [49]. Both country clusters are hence comparable in terms of the gender differences in work volume (around 6 hours/week)—albeit due to different institutional and cultural factors—but a comparison of their LFP rates would suggest that the Scandinavian cluster is the most gender egalitarian. This is evident from its small gender gap in LFP rates (5.6%), driven by progressive parental leave policies, affordable childcare, labor protections that support work-life balance, progressive gender role attitudes, and men’s involvement in unpaid work [54–58]. In contrast, the comparison of average working hours would suggest that the CEE country cluster is more egalitarian, with an average working hour gap of just 3.8 hours per week—presumably shaped by its socialist legacy in terms of state-imposed gender equality and policies promoting full-time employment for all (e.g., through universal childcare access) along with the ongoing economic necessity of dual-earner households [59–62].

A closer examination of both the Scandinavian and Eastern European cluster also reveals quite some within-cluster variation. Out of the five Scandinavian countries, where gender gaps in work volume have declined since the early/mid 1990s, LFP rates rose in three countries (Sweden, Norway, and Finland) but working hours have only grown in one country (Sweden). In Denmark, we even observe declines in both women’s LFP rates and working hours. The developments in the Eastern Europe cluster have been more heterogenous: Even though women’s average working hours declined in all 10 countries, work volume gaps between women and men have declined in six countries (Czech Republic, Hungary, Lithuania, Poland, Romania, Slovenia), but increased in Bulgaria and Estonia and stayed the same in Latvia and the Slovak Republic. This heterogeneity highlights the need for detailed examinations of within-cluster variations in labor market indicators and policy developments and underscores the potential limitations of examining “regimes” and “clusters” rather than single countries [46,59,63,64].

Next, we turn to the countries in the Southern European cluster, where reliance on the family to provide care is high [48,49]. Here, the gender gap in average work volume is relatively moderate, at 8 hours, but it varies considerably across countries, from 4.7 hours in Portugal to 13 in Greece and 12 in Italy. Similar to the Scandinavian countries, the Southern European cluster is characterized by women’s increasing LFP rates and decreasing average working hours over time. In contrast to all other country clusters, however, the developments in women’s LFP rates, average working hours, and work volume (along with the respective gender gaps in LFP rates and work volume) is consistent across countries. Most likely, this is the result from economic pressures and EU requirements for more gender equity [65–67]: Women’s LFP rates and average work volume increased in all six countries while their working hours decreased, yielding declines in gender gaps in both LFP rates and work volume. Changes in gender gaps in average working hours in all six countries are moderate (less than 2 hours) with the exception of Portugal, where we observe a decrease from almost three hours between 1992 and 2022.

Lastly, we turn to the Continental European countries and English-speaking countries and hence the clusters with the largest gender gaps in work volume (country averages of 9.5 and 9 hours/week). Continental European countries are characterized by a legacy of traditional work-family policies, providing families with financial support to care for children rather than infrastructure [48,49]. The Anglo-Saxon regime, by contrast, has been characterized by providing little caregiving support to families [48,49]. Despite these very different policy regimes, gender gaps in work volume decreased considerably in both clusters. This decrease is driven by increases in both women’s LFP rates and average working hours (the only exception being Austria, where women’s weekly working hours have declined since the early 1990s).

## Discussion and conclusion

Gender differences in paid work are multifaceted and stem from various factors, including occupational segregation [68,69], prevailing stereotypes and discrimination [70,71], and the unequal distribution of unpaid care work [72–74]. Disparities in labor force participation rates and working hours are related to all of these sources and particularly relevant to the pursuit of greater gender equality. Not working in paid employment at all or only working a few hours limits women’s economic independence by reducing their current wages, access to social protections, as well as their lifetime earnings and old age pension benefits [5,18,19].

To examine gender differences in paid work over time and between countries, we analyzed harmonized survey data from 849 national probability samples of 32 countries between 1992 and 2022. Having illustrated that the use of either LFP rates or average working hours may lead to distorted results in cross-country and over time comparisons, we introduced the concept of “work volume,” which measures working hours but records hours of zero for those who are not working or are outside of the labor force. We suggest that work volume should be used as a supplementary or alternative measure to LFP rates and average working hours in comparative labor market research.

To highlight the value of the work volume measure in comparing gender equality across countries, let us consider one final example. In 2022, the gender gap in LFP rates was almost five percentage points higher in the United States than in the Netherlands, but the gender gap in employed people’s working hours was more than five hours larger in the Netherlands than the United States. If we relied on these two measures, it would be difficult to say which country had greater gender equality in paid work. Researchers who rely on work volume, however, will find that the gender gap was larger in the Netherlands (10.1 hours) than in the United States (7.7 hours). The work volume measure therefore reveals that, as of 2022, women and men in the United States were more equal in terms of their overall contribution to the workforce than those in the Netherlands.

Our analyses showed that trends in women’s work volume varied greatly across countries and were shaped by countervailing trends in women’s LFP rates and working hours. Women’s LFP rates increased in most countries. In 2022, LFP rates were over 80 percent in most countries (exceptions are Italy, Greece, Romania, and the United States). Women’s average working hours, however, decreased over time in most countries (exceptions are Ireland, Malta, Romania, and the United States, where working hours remained relatively stable, and Sweden, Switzerland, the Netherlands, and the United Kingdom, where hours increased slightly). Over time, men’s work volume either decreased or remained stable (with the exception of Bulgaria and Finland). This trend was driven by men’s decreasing or stagnant LFP rates in most countries and decreasing working hours in all countries. All of these trends led to shrinking gender gaps in work volume, but the remaining gap was still high. In 2022, men’s work volume was, on average, 5.6 hours greater than women’s, and the gender gap in work volume ranged from less than four hours (Lithuania and Bulgaria) to more than 12 hours (Italy and Greece).

When comparing gender gaps in work volume by geographical region and welfare state regime, we found that the former communist Central and Eastern European countries and the social democratic Scandinavian countries had the lowest gender gaps in work volume, but that these relatively low gender gaps were driven by different parameters—a low gap in LFP rates in the Scandinavian countries and a low gap in average working hours in the CEE countries. The conservative Continental European and the liberal Anglo-Saxon regimes had the greatest gender gaps, while the gender gap in the Southern cluster was moderate. Our analyses, moreover, also showed large within-regime variation. Future research should whether the same policies and economic conditions—or their combinations—consistently influence gender gaps in work volume, LFP rates, and working hours to the same extent.

In closing, our comparison of men’s and women’s labor force participation rates, average weekly working hours, and work volume across 32 countries and more than 30 years confirms persistent gender inequalities in paid work in the EU and the US and illustrates how reliance on just one measure may lead to both over- and underestimation of changes over time and of gendered labor market inequalities. The proposed work volume measure can overcome these problems and pitfalls and can also be used for any other type of comparison.

## Appendix

**Fig A1:**
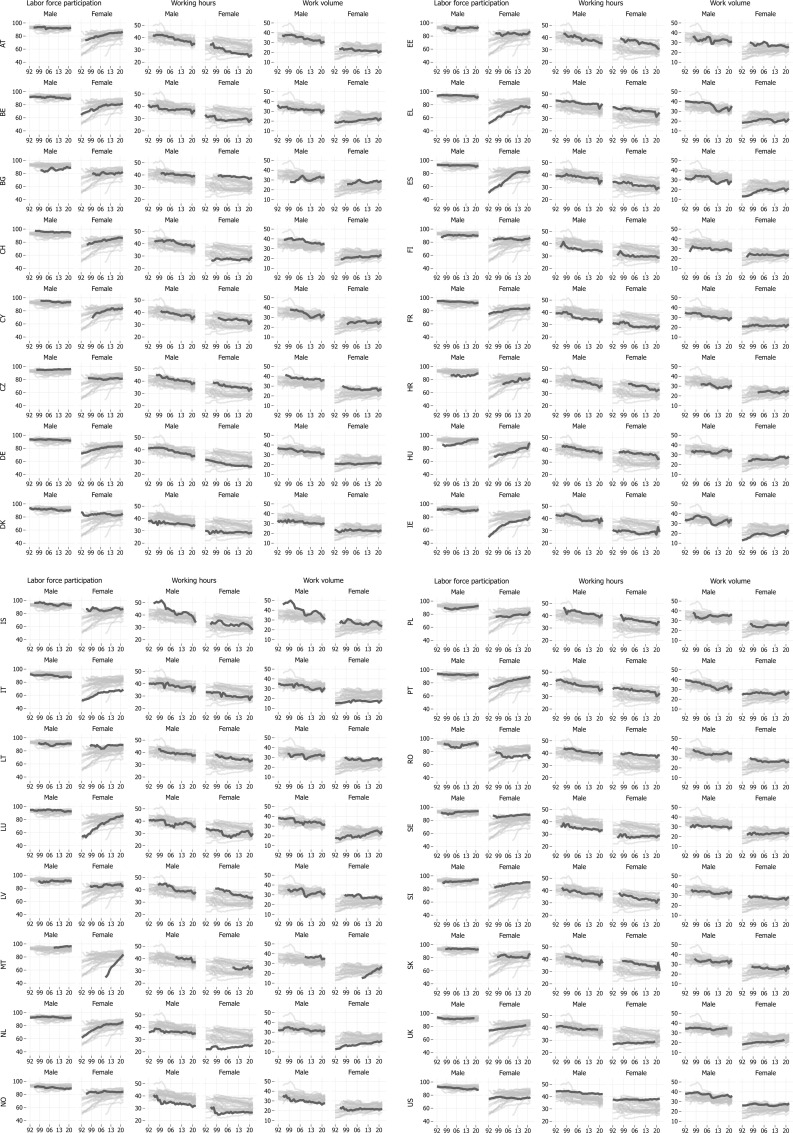
Trends in LFP rates, average working hours, and work volume (in hrs.) in Europe and the United States, 1992–2022. *Notes:* Total N ranges between 38,6 (working hours and work volume) and 38,8 (LFP) million observations; figure is based on individuals aged 25–54 years.
